# 继发于类风湿关节炎的FXIII抑制物所致获得性FXIII缺乏1例

**DOI:** 10.3760/cma.j.cn121090-20250610-00269

**Published:** 2025-09

**Authors:** 炳臣 朱, 华聪 蔡, 炎 张, 立 王, 为 王

**Affiliations:** 1 中国医学科学院北京协和医院血液内科，北京 100730 Department of Hematology, Peking Union Medical College Hospital, Chinese Academy of Medical Sciences, Beijing 100730, China; 2 中国医学科学院北京协和医院全科医学科，北京 100730 Department of General Internal Medicine, Peking Union Medical College Hospital, Chinese Academy of Medical Sciences, Beijing 100730, China

患者，女，75岁，因“左下肢进行性肿痛11 d”于2024年5月16日就诊于北京协和医院。患者2024年5月11日无明显诱因出现左侧大腿肿痛，目测左大腿较右侧肢体肿大，皮肤张力高，皮温高，可见左侧臀外侧约7 cm×8 cm暗紫色淤青；2 d后左侧肢体（大腿、小腿及左脚）肿痛较前加剧，较右侧肢体明显肿大，局部红肿、皮温升高，皮肤张力高，触痛明显，行动不能，伴嗜睡。

既往史：类风湿关节炎（RA）20余年，间断口服中药治疗，此次病来无关节痛、晨僵；白癜风病史16年；青霉素、磺胺药物过敏。既往无出血史；个人史、婚育史、家族史无特殊，无近亲结婚。

查体：体温36.5 °C，血压121/79 mmHg（1 mmHg＝0.133 kPa），SpO_2_: 93％，体重指数23.72 kg/m^2^，腹部膨隆，全身皮肤可见散在色素缺失，睑结膜苍白，口唇苍白，双下肺呼吸音粗，可闻及Velcro爆裂音，腹部质韧，无压痛、反跳痛，肠鸣音活跃，6～8次/min，左侧肢体可见肿胀，按压疼痛，皮肤张力较高，皮温高，左侧小腿围（膝盖下10 cm）34.5 cm，左侧大腿围（膝盖上18 cm）49.4 cm，右侧小腿围（膝盖下10 cm）30.1 cm，右侧大腿围（膝盖上18 cm）41.9 cm。左侧肢体、会阴区、双侧臀部、腰背部可见大片淤青。双手、腕关节无畸形、活动受限，各指间关节无按压痛。

实验室检查：WBC 10.85×10^9^/L，HGB 54 g/L，PLT 107×10^9^/L；血白蛋白（Alb）28 g/L，总胆红素39.8 µmol/L，直接胆红素14.9 µmol/L；超敏C反应蛋白（hsCRP）51.0 mg/L，红细胞沉降率（ESR）35 mm/1 h；凝血酶原时间（PT）12.3 s，活化部分凝血活酶时间（APTT）24.3 s，纤维蛋白原（Fbg）2.72 g/L，D-二聚体（D-Dimer）1.37 µg/L；纤维蛋白降解产物（FDP）6.94 µg/ml；vWF抗原（vWF-Ag）236.4％；凝血因子XIII（FXIII）定性：阳性（患者柠檬酸盐血浆凝块20 min完全溶解，正常对照血浆凝块24 h未溶解，纠正试验血浆凝块溶解，提示FXIII抑制物存在）；FXIII纠正试验：1∶1正常血浆不可以纠正；大腿MRI：左大腿股四头肌及股内侧肌群肿胀，股直肌内见约11.0 cm×7.5 cm×3.0 cm巨大团块状混杂信号影，左股骨干髓腔内及软组织异常信号，股直肌脓肿、出血。

结合患者实验室检查结果，考虑获得性F??抑制物明确，病初患者HGB水平进行性下降，予以下肢血管造影+栓塞术紧急止血，术后对症输注红细胞、血浆支持，监测HGB逐步上升；稳定期予以利妥昔单抗500 mg静脉滴注（第0天）+硼替佐米2 mg皮下注射（第1、4、8、11天）治疗，但过程中患者反复发热，感染筛查阴性，抗感染治疗无效，反复查炎症指标高，因患者存在RA病史，遂完善相关检查：ANA抗体谱1∶80；类风湿因子（RF）88.8 lU/ml，抗环瓜氨酸多肽抗体（抗CCP）2 960 U/ml，抗MCV抗体714 RU/ml，补体3（C3）0.40 g/L，补体4（C4）0.07 g/L；免疫科会诊考虑RA活动明确，加用甲泼尼龙40 mg每日1次静脉滴注×3 d（后改为泼尼松20 mg每日2次口服），环孢素75 mg每日2次治疗后患者未再发热，出血情况稳定，考虑为继发于RA的FXIII抑制物。2024年8月15日复查FXIII定性试验仍阳性，WBC 8.37×10^9^/L，HGB 78 g/L，PLT 115×10^9^/L，考虑合并慢性病贫血，给予EPO治疗。目前患者规律随访中。

讨论：RA相关FXIII抑制物的报道罕见，本例进一步丰富了此类罕见并发症的临床特征和治疗经验。对于不明原因深部出血且PT、APTT、TT、Fbg正常者，需警惕FXIII先天性或获得缺乏可能。继发因素中，尽管系统性红斑狼疮占主导，但RA等非典型免疫病亦不容忽视。治疗需兼顾止血、抑制抗体生成及控制原发病，多学科协作可改善患者预后。

